# Association of HLA genotypes, AB0 blood type and chemokine receptor 5 mutant CD195 with the clinical course of COVID-19

**DOI:** 10.1186/s40001-021-00560-4

**Published:** 2021-09-16

**Authors:** Johannes C. Fischer, Albrecht G. Schmidt, Edwin Bölke, Markus Uhrberg, Verena Keitel, Torsten Feldt, Björn Jensen, Dieter Häussinger, Ortwin Adams, E. Marion Schneider, Vera Balz, Jürgen Enczmann, Jutta Rox, Derik Hermsen, Karin Schulze-Bosse, Detlef Kindgen-Milles, Wolfram Trudo Knoefel, Martijn van Griensven, Jan Haussmann, Balint Tamaskovics, Christian Plettenberg, Kathrin Scheckenbach, Stefanie Corradini, Alessia Pedoto, Kitti Maas, Livia Schmidt, Olaf Grebe, Irene Esposito, Anja Ehrhardt, Matthias Peiper, Bettina Alexandra Buhren, Christian Calles, Andreas Stöhr, Artur Lichtenberg, Noemi F. Freise, Matthias Lutterbeck, Amir Rezazadeh, Wilfried Budach, Christiane Matuschek

**Affiliations:** 1 Institute for Transplantation Diagnostics and Cell Therapeutics, University Hospital Dusseldorf, Medical Faculty, Heinrich-Heine-University, 40225 Dusseldorf, Germany; 2 Department of Radiation Oncology, University Hospital Dusseldorf, Medical Faculty, Heinrich-Heine-University Dusseldorf, Moorenstr. 5, 40225 Dusseldorf, Germany; 3 Department of Gastroenterology, Hepatology and Infectious Diseases, University Hospital Dusseldorf, Medical Faculty, Heinrich-Heine-University Dusseldorf, Moorenstr. 5, 40225 Dusseldorf, Germany; 4 Institute for Virology, University Hospital Dusseldorf, Medical Faculty, Heinrich-Heine-University Dusseldorf, Universitaetsstr. 1, 40225 Dusseldorf, Germany; 5 Division of Experimental Anesthesiology, University Hospital Ulm, Ulm, Germany; 6 Central Institute for Laboratory Diagnostics and Clinical Chemistry, Medical Faculty Heinrich-Heine University, Dusseldorf, Germany; 7 Medical Faculty, Department of Anesthesiology, Heinrich Heine University, Dusseldorf, Germany; 8 Medical Faculty, Department of Surgery and Interdisciplinary Surgical Intensive Care Unit, Heinrich Heine University, Dusseldorf, Germany; 9 Department cBITE, Maastricht University, MERLN Institute for Technology-Inspired Regenerative Medicine, Maastricht, the Netherlands; 10 Medical Faculty, Department of Ear, Nose and Throat Disease, Heinrich Heine University, Dusseldorf, Germany; 11 Department of Radiation Oncology, University Hospital, LMU Munich, Munich, Germany; 12 Department of Anesthesiology, Memorial Sloan Kettering Cancer Center, New York, NY USA; 13 Department of Cardiology and Rhythmology, Petrus Hospital, Wuppertal, Germany; 14 Institute of Pathology, University of Dusseldorf, Dusseldorf, Germany; 15 Institute of Virology, University of Witten/Herdecke, Witten, Germany; 16 Medical Faculty, University of Dusseldorf, Dusseldorf, Germany; 17 Medical Faculty, Coordination Center for Clinical Studies, University of Dusseldorf, Dusseldorf, Germany; 18 Department of Cardiac Surgery, Medical Faculty, University of Dusseldorf, Dusseldorf, Germany

**Keywords:** SARS-CoV-2, HLA class I genotypes, ABO blood group, Chemokine receptor, CCR5, Neutralizing antibodies, COVID-19, Clinical course

## Abstract

**Background:**

COVID-19, the pandemic disease caused by infection with SARS-CoV-2, may take highly variable clinical courses, ranging from symptom-free and pauci-symptomatic to fatal disease. The goal of the current study was to assess the association of COVID-19 clinical courses controlled by patients’ adaptive immune responses without progression to severe disease with patients’ Human Leukocyte Antigen (HLA) genetics, AB0 blood group antigens, and the presence or absence of near-loss-of-function delta 32 deletion mutant of the C–C chemokine receptor type 5 (CCR5).

**Patient and methods:**

An exploratory observational study including 157 adult COVID-19 convalescent patients was performed with a median follow-up of 250 days. The impact of different HLA genotypes, AB0 blood group antigens, and the CCR5 mutant CD195 were investigated for their role in the clinical course of COVID-19. In addition, this study addressed levels of severity and morbidity of COVID-19. The association of the immunogenetic background parameters were further related to patients’ humoral antiviral immune response patterns by longitudinal observation.

**Results:**

Univariate HLA analyses identified putatively protective HLA alleles (HLA class II DRB1*01:01 and HLA class I B*35:01, with a trend for DRB1*03:01). They were associated with reduced durations of disease instead decreased (rather than increased) total anti-S IgG levels. They had a higher virus neutralizing capacity compared to non-carriers. Conversely, analyses also identified HLA alleles (HLA class II DQB1*03:02 und HLA class I B*15:01) not associated with such benefit in the patient cohort of this study. Hierarchical testing by Cox regression analyses confirmed the significance of the protective effect of the HLA alleles identified (when assessed in composite) in terms of disease duration, whereas AB0 blood group antigen heterozygosity was found to be significantly associated with disease severity (rather than duration) in our cohort. A suggestive association of a heterozygous CCR5 delta 32 mutation status with prolonged disease duration was implied by univariate analyses but could not be confirmed by hierarchical multivariate testing.

**Conclusion:**

The current study shows that the presence of HLA class II DRB1*01:01 and HLA class I B*35:01 is of even stronger association with reduced disease duration in mild and moderate COVID-19 than age or any other potential risk factor assessed. Prospective studies in larger patient populations also including novel SARS-CoV-2 variants will be required to assess the impact of HLA genetics on the capacity of mounting protective vaccination responses in the future.

**Supplementary Information:**

The online version contains supplementary material available at 10.1186/s40001-021-00560-4.

## Introduction

Genetic factors underlying the wide inter-individual variability of the clinical course of COVID-19 remain poorly characterized to date. Most clinical studies dissecting the association of genetic characteristics with COVID-19 focus on either the susceptibility to infection or on disease progression to serious morbidity or mortality. In the current study, we took the diverging approach of assessing immunogenetic characteristics for their association with COVID-19 clinical courses as well as with adaptive antiviral humoral immune response patterns in patients who mastered the infection without progression to severe manifestations (WHO° 3 or less).

Our analyses focused on immunogenetic background characteristics governing adaptive antiviral immune responsiveness and durable maintenance thereof in different ways:

First, we were interested in elucidating the potential impact of CCR5 delta32 (CD195), a near-loss-of-function mutant of the C–C chemokine receptor type 5 on the COVID-19 clinical course. CCR5 has been associated with susceptibility or resistance to a broad spectrum of viral diseases [1–3]. Further, a recent epidemiological analysis suggested that the CCR5 delta32 mutation is associated with increased susceptibility to SARS-CoV-2 infection and fatal COVID-19 outcome [4]. CCR5 is known to play a decisive role as a chemotactic receptor abundantly expressed on monocytes, macrophages and T-cells and its heterozygous mutation-related deficiency implies impaired memory CD4 + T-cell response [5]. Thus, in regard to the clinical course of COVID-19, the roles of CCR5 (1) within the oral–pharyngeal immune system as the first line of antiviral immune defense and (2) as a critical receptor governing adaptively induced T-cell memory provide a compelling immune-mechanistic rationale for the putative association of the delta32 near-loss-of-function mutation of CCR5 with altered susceptibility to SARS-CoV-2 infection and COVID-19 morbidity [6–9].

Second, we analyzed the HLA genotypes of our patient cohort. Recognition of viral antigens presented on HLA molecules to T-cell receptors is central to all T-cell mediated antiviral immune responses, both cellular cytotoxic and humoral [10]. Thus, HLA haplotypes directly impact the clonal architectures of T-cell dependent adaptive immune responses targeting SARS-CoV-2. Therefore, they play a crucial role in shaping T- and B-cell clonal architectures. With this knowledge it is not surprising that distinct HLA alleles have been found to be associated with increased or decreased SARS-CoV-2 susceptibility and COVID-19 morbidity, respectively [11–14].

In addition, the current study also considered the potential impact of AB0 blood group antigens assessed at genetic level on COVID-19 morbidity. Blood group antigens were included in our genetic data sets because blood group A (genotype AA or A0) has been shown to be associated with severe disease (WHO° 5–8) in a genome wide association study [9]. However, the precise level of COVID-19 risk strictly attributable to AB0 blood group antigens per se (rather than linked co-variates, such as isoagglutinin levels or Rhesus system antigens) remains a subject of uncertainty following study outcomes assigning different levels of risk [15–17].

## Patients, materials and methods

The study was performed according to the declaration of Helsinki. The local ethics committee, University of Düsseldorf, Germany (registration and approval number 2020–1116) authorized the study protocol. As stipulated by the study protocol, genomic AB0 blood groups, HLA-A*, -B*, -C*, -DRB1*, -DQB1*, and -DPB1* genotypes, and presence vs. absence of the C–C chemokine receptor type 5 (CCR5) delta 32 mutation were determined in patients consenting to the study procedures as well as to potential donation of convalescent plasma.

Key inclusion criteria for potential convalescent plasma donors were: age between 18 to 65 years, and SARS-CoV-2 infection between 17th of February 2020 and 27th April 2020, confirmed by PCR before study enrollment. In addition, written informed consent had to be given.

Key exclusion criteria were age under 18 years, inability to give informed consent, pregnancy or breast-feeding, chronic infections including viral hepatitis and HIV infections, and malignancies before enrollment.

End points of our study were antibody response and duration of disease.

The study cohort consisted of 78 male (mean age of 42.9 ± 13.3 years, range 19.7–76.8 years) and 79 female potential plasma donors (mean age of 45.3 ± 13.7 years, range 19.9–79.5 years). Characteristics of the patients can be found in Table 1. Severity of disease was classified as 0 for patients without any symptoms (corresponding to WHO COVID-19 ordinal scale WHO°1), as 1 in patients with only mild symptoms and no relevant restriction of activities (WHO°1), 2 for patients with restriction of activities (corresponding to WHO°2a) and 3 for patients with more severe symptoms (corresponding to WHO°2b and borderline WHO°3). None of the patients was hospitalized. In symptomatic patients activity restriction was confirmed by telephone interview.

**Table 1:** Patient’s characteristics

	Male	Female
Number	*n* = 78	*n* = 79
Age	42.9 ± 13.3 (19.7–76.8 years)	45.3 + 13.7 (19.9–79.5 years)
Mild disease	27	26
Moderate disease	51	53
Severe disease	0	0
Mortality	0	0

### Antibody assessments

To assess the antibody response, samples were tested for IgA and IgG antibodies against the spike S1 protein using the anti-SARS-CoV-2-ELISA (IgA) and anti-SARS-CoV-2-ELISA (IgG) (Euroimmun AG, Lübeck, Germany). Total Ig level of antibodies against the nucleocapsid were determined by the Elecsys® Anti-SARS-CoV-2 (Roche, Rotkreuz, Switzerland) confirmatively. In addition, the IgG antibody levels against virus nucleocapsid antigen (Euroimmun) were measured appropriate immune globulins in donors. All assays were conducted according to manufacturer’s standard operating procedures/ protocols.

### Genotyping for HLA, AB0 Blood group and CCR5 wild type (wt) assessment

An amplicon-based next generation sequencing (NGS) approach was used as described before [18, 19]. Briefly, amplification of exons 2, 3, and 4 of HLA class I and HLA-DPB1 genes, and exons 2 and 3 of HLA-DRB1 and HLA-DQB1 (in parallel with selected exons of the blood group system genes AB0 and Rh as well as exon 3 of the CCR5 gene for detection of CCR5 wt or CCR5 del32 or other mutations causing a truncated CC chemokine receptor type 5) were carried out in six multiplex polymerase chain reactions (PCRs). After a purification step using paramagnetic beads, the amplificates underwent a second-round PCR that added sample-specific barcodes and Illumina-compatible adapter sequences. All PCR products were pooled, the resulting library was quantified using real-time PCR applying primers directed to the Illumina-specific adapter (Illumina Inc., San Diego, CA) and were sequenced on a MiSeq device (Illumina). The analysis of the read sequences was performed by an in-house software (NGSSequence Analyser, Institute of Transplantation Diagnostics and Cell Therapeutics, University Hospital of Düsseldorf, Düsseldorf, Germany) approach taking into account quality control values and high coverage to automate data analysis. Sophisticated algorithms were developed to distinguish between sequencing artefacts, such as cross-over products and closely related alleles, as well as for the identification of novel alleles.

To determine the complete HLA gene sequence in questionable results, we developed a second NGS work flow. After amplification of the HLA gene using primers directed to the 5′- and 3′ untranslated regions (UTRs), the amplification was fragmented, end-polished and ligated to Y-adapters. A second PCR supplemented the resulting fragments with sample-specific barcodes and Illumina compatible adapters. The samples in test were pooled, the obtained library was quantified using a real-time PCR approach and run on a MiSeq instrument (Illumina). In case of uncertain results, the alignment of the reads and the analysis were performed using the NGSengine software (GenDx, Utrecht, The Netherlands).

### Neutralizing antibodies testing

Donor plasma or sera were heat-inactivated for 30 min at 56 °C. Twofold serial dilutions (ranging from 1:5 to 1:10,240) were prepared in 50 µl volume of maintenance medium (Dulbecco’s Modified Eagle Medium (Thermo Fisher), 100 U/mL Penicillin and 100 μg/mL Streptomycin (Gibco), 2% Fetal Calf Serum (PAN Biotech)). All samples were tested in duplicate. Then, 50 µl of the virus stock solution with the SARS-CoV-2 NRW-42 isolate was added to a final concentration of 280 Tissue Culture Infection Dose 50 (TCID50)/ml. Virus stock was stored at -80 °C and the infectious viral titer was determined every 4 weeks. Cell-free plates were pre-incubated at 37 °C, 5% CO_2_ for 1 h before 100 µl of cell suspension containing 7 × 10^4^/ml Vero cells (ATCC-CCL-81, obtained from LGC Standards), continuously checked for mycoplasma contamination and added to the donor samples. Plates were then incubated at 37 °C, 5% CO_2_ for 96 h.

The serum neutralization titer was determined by microscopic inspection as the highest serum dilution without virus-induced cytopathic effect (CPE). As positive controls, two previously tested sera from SARS-CoV-2-infected individuals were tested at the same time in each run. One high-titer control (NT 1:640) and one medium-titer control (NT 1:160) were used for validation of each assay. Inter-assay and intra-assay variation were determined with these control sera showing a maximum deviation of only one dilution step. In addition, serum from SARS-CoV-2-uninfected individuals and cells without serum served as negative controls to confirm virus-induced CPE. Omission of serum and virus solution served as control for cell growth during the conducted protocol.

A neutralization titer of 1:160 was defined as the threshold titer for binary representations of virus neutralization capacity allowing the potential plasmapheresis product to serve as a therapeutic agent. As the protocol reports the highest serum/plasma dilution with neutralization of 100% of infectious virus particles, the reported titers are a conservative estimate of the neutralizing capacity of donor plasma. The neutralization titers of 118 donors with a confirmed positive test result for SARS-CoV-2 by PCR or a reported episode of symptoms consistent with COVID-19 and a positive result in serological testing for anti-SARS-CoV-2 were tested in the current study.

### Statistics

Statistical inference between two groups of interest was assessed by *t* test statistics. Comparison of duration-of-infection times between groups of interest was performed by Kaplan–Meier curves and with two-sided log rank tests. Multivariable Cox regression analysis was performed to identify significant predictors for duration-of-infection. Effects in the multivariate Cox regression models were quantified by hazard ratio estimates with corresponding 95% confidence intervals. All *p* values were calculated with two-sided tests and *p* values less than 0.05 were considered statistically significant. Analyses were conducted using IBM SPSS Statistics for Windows (Version 22.0. Released 2013, Armonk, NY: IBM Corp) as well as R (3.6.3, https://www.r-project.org/).

## Results

This exploratory observational study enrolled a total of *n* = 157 adult Caucasian COVID-19 convalescent patients, who were followed-up in an out-patient setting. Patient-age ranged from 20 to 79.5 years (mean age 44 years) with a balanced distribution of female (*n* = 79) versus male (*n* = 78) patients.

Study participants had suffered and recovered from mild to moderate COVID-19 disease, whereby approximately 38% of patients had experienced mild (WHO° 1 and 2a) and 52% moderate COVID-19 disease (WHO° 2b and 3). Patients who had experienced mild disease were younger (age range 19.7–79.5 years, mean 41.2 years, median 38.3 years) than patients experiencing moderate disease (age range 20.7–66.5 years, mean 45.2 years, median 49.6 years, *p* < 0.028). No other notable differences in terms of demographic characteristics, comorbidities or concomitant medications were observed between cohorts recovered from different severity grades of COVID-19. This statement applies to all sub-group analyses reported here, unless specifically noted otherwise.

**Fig. 1:**
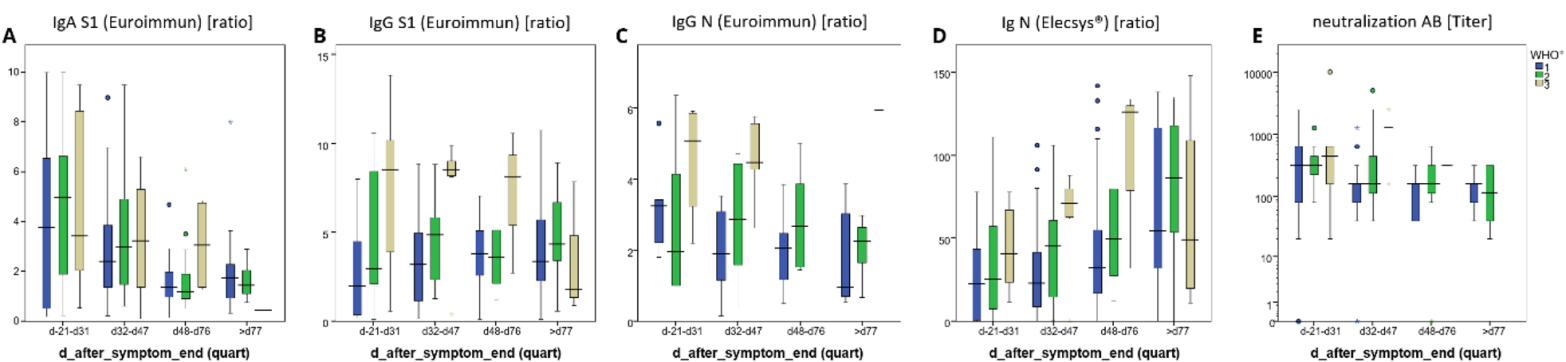
SARS-CoV-2 viral protein-specific antibody levels in plasma drawn up to 250 days following the end of the symptoms from individuals with different disease severity. Disease severity was according to WHO classification (Blue indicates WHO °1, green WHO °2, gold WHO °3). Shown are the results of the first blood draw after including the individual into the study. **A** S1 protein-specific IgA antibody levels, measured with the Euroimmun assay. Results are expressed as ratio. **B** S1 protein-specific IgG antibody levels, measured with the Euroimmun assay. Results are expressed as ratio. **C** Nucleocapsid (N)-specific IgG antibody levels measured with the Euroimmun assay. Results are expressed as ratio. **D** N-specific Ig antibody levels detected with Elecsys®, Roche. **E** The SARS-CoV-2 serum neutralization titer, determined by microscopic inspection as the highest serum dilution without virus-induced cytopathic effect. Outliers are presented as circles (more than 1.5 IQR out of the box) or stars (more than 3 IQR out of the box). Day after end of symptoms was divided into quartiles (d20 to d31, d32-d47, d48-d76, > d77, range between d20 to d120)

In this convalescent patient cohort, S1-specific IgA antibody levels were barely detectable (ratio < 0.8) or low (ratio 0.8–1.0) in 41 of the 130 tested individuals yet markedly elevated (ratio ≥ 1.1) in the remaining 89 patients. IgA antibody levels were lower in blood samples taken later than 32 days following the end of symptoms (Fig. 1a). These IgA antibody kinetics are principally in line with earlier longitudinal analyses of IgA responses against SARS-CoV-2 antigens [20, 21]. No apparent correlation between IgA antibody titers and severity of disease was observed in our study population as a whole (Fig. 1a).

**Fig. 2:**
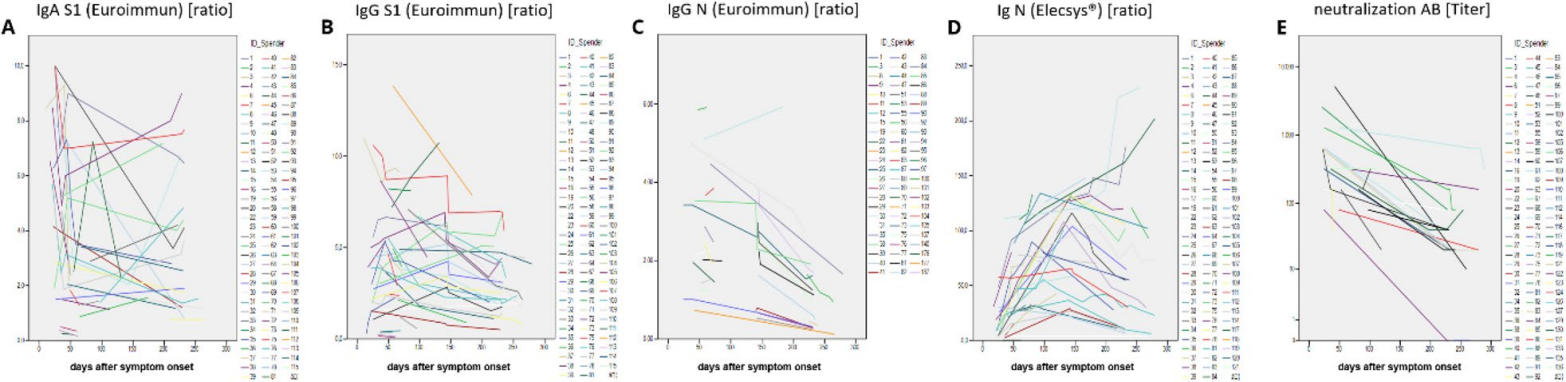
SARS-CoV-2 viral protein-specific antibody levels in plasma samples sequentially drawn from infected individuals between day 10 and day 188 following symptom onset. SARS-CoV-2 viral protein-specific antibody levels were determined as described in the legend of Fig. 1

S1-(Fig. 1b) and N- (Fig. 1c) specific IgG antibody levels were scattered over a large concentration range over the whole study period. However, in contrast to anti-S IgA levels, anti-S IgG titers were higher in individuals with more severe disease (Fig. 1b and d), in accordance with findings reported before [21–23]. Throughout the study observation period of up to 240 days after the end of symptoms, IgG antibody levels tended to be stable or even elevated in 29 of the 42 subjects tested, whereas they decreased in the remaining 13 subjects (Fig. 2b, c). In all patients in whom elevated antiviral IgG antibodies had been observed at earlier timepoints, the respective anti-S IgG antibodies remained detectable at the last observation recorded.

Of note, 81 of 118 samples analyzed in this study (68.6%) neutralized virus at the prospectively defined minimum threshold titer of 1:160 or higher at time point of enrollment into the study. This proportion of samples exhibiting relevant virus neutralizing capacity is in line with results of a previous study in a similar cohort of convalescent COVID-19 patients [21]. While a certain level of correlation between the two parameters neutralizing antibody titers and anti-S IgG levels is found (*R*^2^ = 0.299.), the wide interindividual scatter allows for the conclusion that total anti-S IgG levels are not adequately predictive of virus neutralizing capacity (data not shown). Thus, at the individual patient level, total ELISA determined anti-S IgG levels do not permit to reliably discern high or low levels of neutralizing anti-S IgG antibodies.

Sensitivity analyses for patients’ gender and age did not identify any statistically significant and clinically meaningful differences between the respective cohorts, neither in regard to antiviral antibody titers nor their kinetics (Additional file 1: Fig S1). For a potential exception, a consistent trend in younger patients (< 32 years of age) towards lower antiviral antibody titers compared to older patients was noted, potentially indicative of a more prominent role of adaptive T-cell mediated rather than humoral antiviral immune responses in younger patients and/or anti-S IgG antibody epitope specificities of higher protective capacity yet the trend was not highly significant. The trend nevertheless affirms that low anti-S antibody titers cannot be readily interpreted as lack of antiviral protection, specifically when observed in younger patients. This interpretation was supported by the distribution of antiviral sero-negative patients across age groups.

**Fig. 3:**
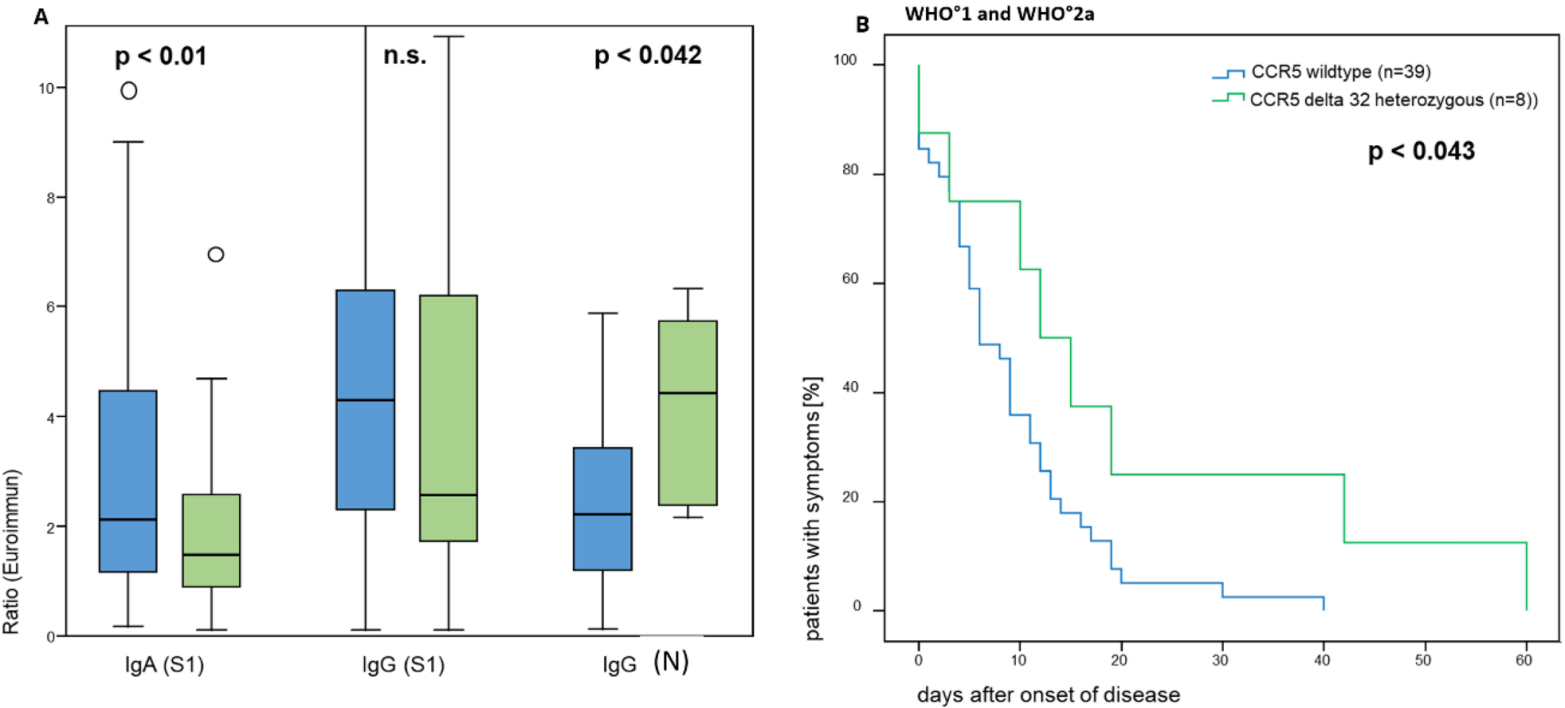
Significantly lower SARS-CoV-2 viral protein-specific antibody levels and significantly longer disease duration in individuals carrying the CCR5 delta 32 mutation heterozygously with WHO° 1 and WHO° 2a disease following SARS-CoV-2 infection. SARS-CoV-2 viral protein-specific antibody levels were determined as described in the legend of Fig. 1 in 23 individuals carrying the CCR5 delta 32 mutation heterozygously and in 105 individuals carrying the wild type CCR5 gene (**A**). Disease duration in individuals with WHO° 1 and WHO° 2a disease following SARS-CoV-2 infection is shown in **B** for 8 heterozygous carriers of the CCR5 delta mutation and 39 non carriers

Next, we analyzed the impact of the CCR5 delta 32 mutation on COVID-19 morbidity and the humoral antiviral immune response in 126 of the 157 individuals. The median concentrations of S1-specific IgA antibodies and of N-specific IgG antibodies were lower (*p* < 0.01) respectively higher (*p* < 0.042) (Fig. 3a) in the 23 heterozygous carriers of the CCR5 delta 32 mutation, as compared to the 103 tested individuals carrying wild type CCR5. However, S1-specific IgG antibody levels did not differ between carriers of the CCR5 delta 32 mutation as compared to carriers of only CCR5 wild-type alleles (Fig. 3a), while duration of disease was prolonged significantly according to univariate analysis (*p* < 0.04) in the presence of the CCR5 delta 32 mutation (Fig. 3b). Our data do not provide evidence that the heterozygous presence of the CCR5 delta 32 mutation is associated with more severe disease, i.e., we did not find this mutation over-represented in patients with more severe disease, with 8 of 23 carriers of the mutation (35%) experiencing mild disease.

**Fig. 4:**
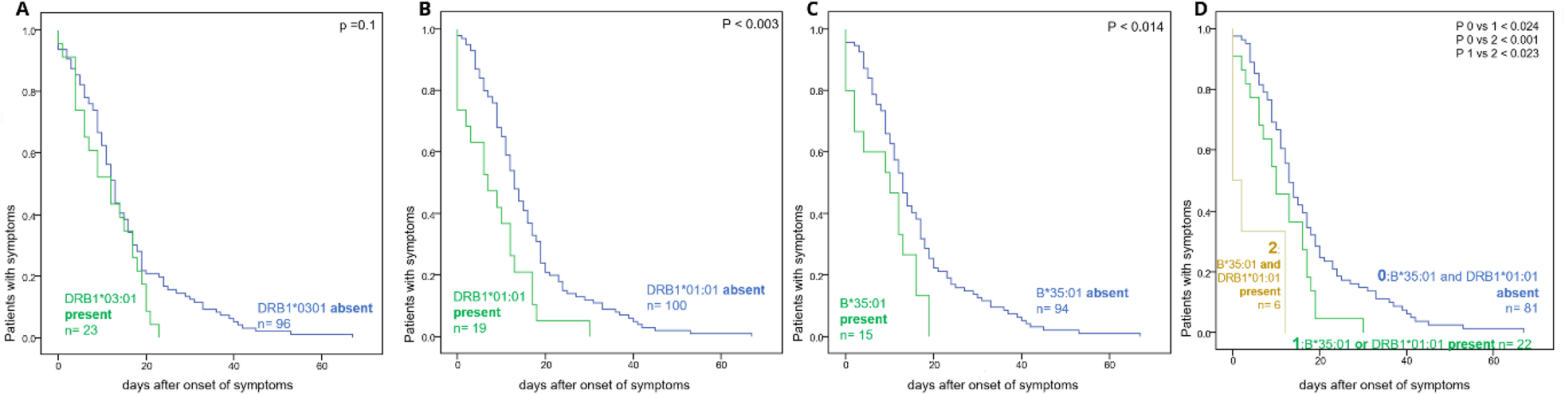
Association of HLA-DRB1*03:01, HLA-DRB1*01:01 and/ or HLA-B*35:01 allele expression with shorter COVID19 disease duration in SARS-CoV-2 infected individuals.** A** The disease duration related to HLA DRB1* 03:01 expression, **B** The disease duration related to HLA*DBR1*01:01 expression, **C** Disease duration related to HLA B* 35:01 expression. **D** The disease duration related to the combination of HLA: DRB1*01:01 and or HLA B* 35:01 expression. (0) indicates the 81 individuals who express neither allele, (1) indicates the 22 individuals who express HLA: DRB1*01:01 (*n* = 13) or HLA B* 35:01 allele (*n* = 9), and (2) indicates the 6 individuals expressing both alleles

Previously, the HLA class II allele DRB1*03:01 has been reported to be associated with a potentially more favorable outcome of SARS-CoV-1 in two Asian populations [12–14]. In this study, where 119 Caucasian individuals were HLA typed, we also observed that those 20 of them expressing the DRB1*03:01 allele tended to have shorter disease durations. However, the difference did not reach statistical significance (Fig. 4a).

The HLA class II allele DRB1*01:01 (Fig. 4b) and the HLA class I allele B*35:01 (Fig. 4c), which were expressed in 19 and 15 individuals, respectively, were associated with a significantly shorter disease duration (*p* < 0.003 and *p* < 0.014, respectively). The disease duration was even shorter in the six individuals expressing two of the identified “protective” HLA alleles (Fig. 4d). However, 18 of the individuals expressing the DRB1*01:01 allele co-expressed the HLA-DQA1*01:01 DQB1*05:01 allele. Therefore, the association of one or both alleles with disease duration remains to be determined.

It is noteworthy that the “protective” HLA alleles share the characteristic of binding a large number of the peptides putatively derived from 9621 SARS-CoV-2 viral proteins at a high cumulative calculated HLA-allele affinity score by “in silico” analysis [24].

In this anaylsis DRB1*01:01, DRB1*03:01 and DQA1*01:01 DQB1*05:01 putatively bind 1758, 997 and 837 of the 7903 HLA class II binding peptides presumably derived from the SARS-CoV-2 viral proteins at a cumulative calculated HLA allele affinity score of at least 1422, 1035 and 918, respectively. In comparison the HLA class I allele B*35:01 putatively binds 183 of the 3540 HLA class I binding peptides presumably derived from the SARS-CoV-2 viral proteins at a cumulative calculated HLA allele affinity score of at least 46.8.

In contrast, the disease durations tended to be longer (*p* < 0.061 and < 0.095, respectively, Additional file 1: Fig S2) in individuals who expressed the HLA class II allele DQB1*03:02 or the HLA class I allele B*15:01. The latter two alleles share the characteristic of binding a low number of the peptides derived from the SARS-CoV-2 viral protein at a low cumulative calculated HLA allele affinity score. Specifically, the B*15:01 allele would only bind 75 of the 3540 HLA class I binding SARS-CoV-2 peptides at a cumulatively calculated HLA allele affinity score lower than 21.8. The DQB1*03:02 allele putatively binds 574 of the 7903 HLA class II binding SARS-CoV-2 peptides at a cumulatively calculated HLA-allele affinity score lower than 619.

**Fig. 5:**
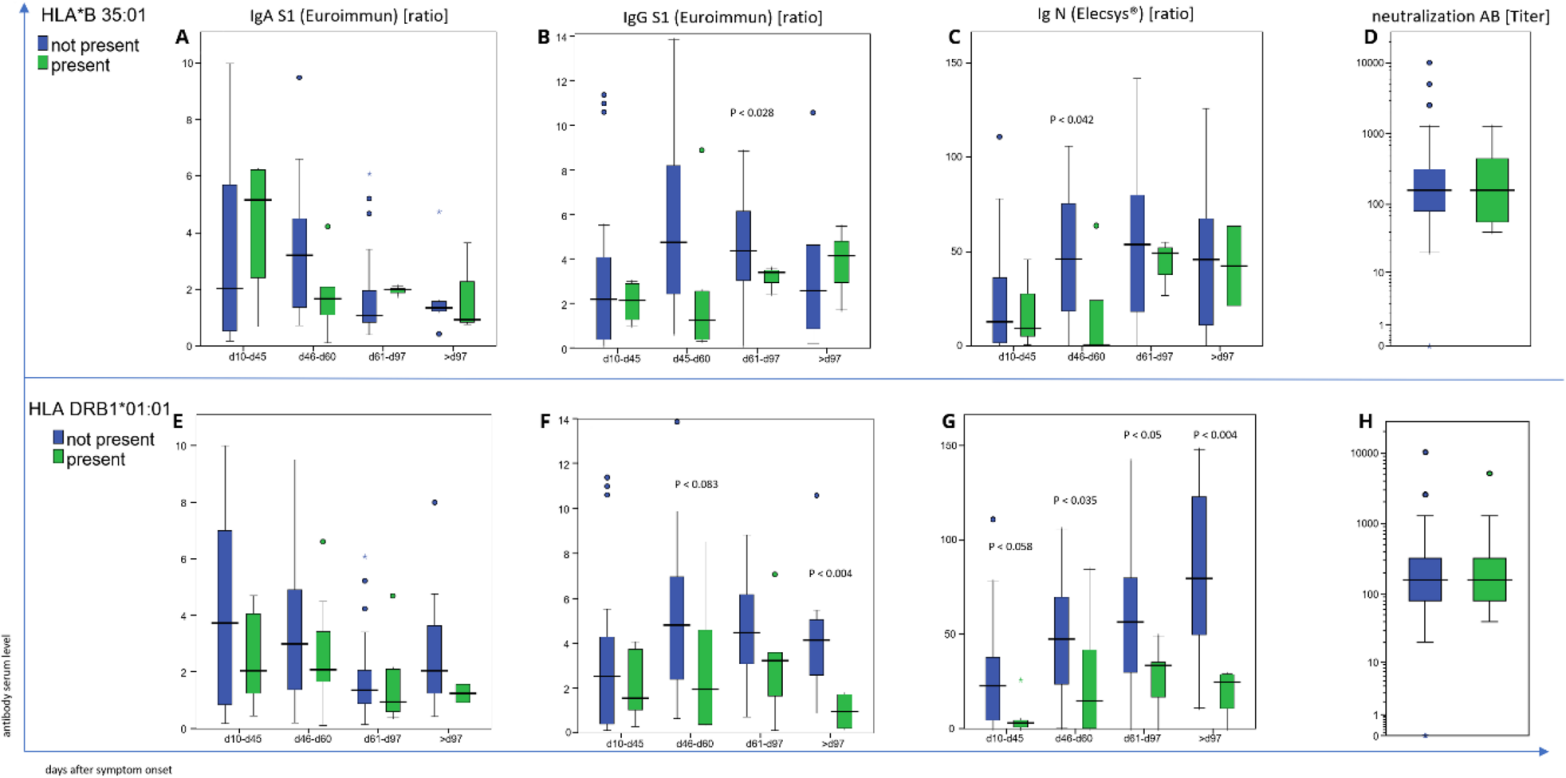
Association of “protective” HLA alleles with SARS-CoV-2 viral protein-specific antibody levels. **A** and **E** show similar levels of S1-specific IgA antibodies in the individuals who express “protective” HLA alleles and those who do not. **B** and **C** show a significantly lower level of S1-specific IgG antibodies and N-specific antibodies in the 15 individuals who express the HLA-B*35:01 allele (green) than in the 94 individuals who do not express (blue). **F** and **G** show a significantly lower level of S1-specific IgG antibodies and N-specific antibodies in the 19 individuals who express the HLA-DRB1*01:01 allele (green) than in the 100 individuals who do not express (blue). **D** and **H** show no detectable difference in the titer of neutralizing antibodies between individuals who express “protective” HLA alleles and those who do not

No significant differences in the titer of neutralizing antibodies (Fig. 5d, i) or in the plasma concentration of S1-specific IgA antibodies (Fig. 5a, f) were found between the groups of individuals expressing the “protective” HLA alleles and of those individuals who did not. In contrast, the plasma concentrations of the S1-specific IgG (Fig. 5 b, g) and N-specific (Fig. 4c, h) antibodies were significantly lower in the group of individuals who expressed the “protective” HLA alleles (*p* < 0.028—< 0.004, < 0.035—0.004 depending on the timepoint after symptom onset, respectively). This finding in conjunction with the lack of difference in the titer of neutralizing antibodies suggests that individuals expressing “protective” HLA alleles produce antibodies with higher avidity.

Blood group antigens are the third immunogenetic category analyzed in this study. The blood group A (genotype AA or A0) has been recently shown to be associated with severe disease (WHO° 5–8) [9]. Furthermore, AB0 blood group isoagglutinins have been shown to inhibit S-protein-ACE2 interactions [25]. In our cohort, the agglutination rate of incompatible blood group erythrocytes was higher in the 40 homozygous 00 individuals and in the 10 homozygous AA individuals than in the 54 heterozygous A0, 13 heterozygous B0 and in the 5 AB individuals.

**Fig. 6:**
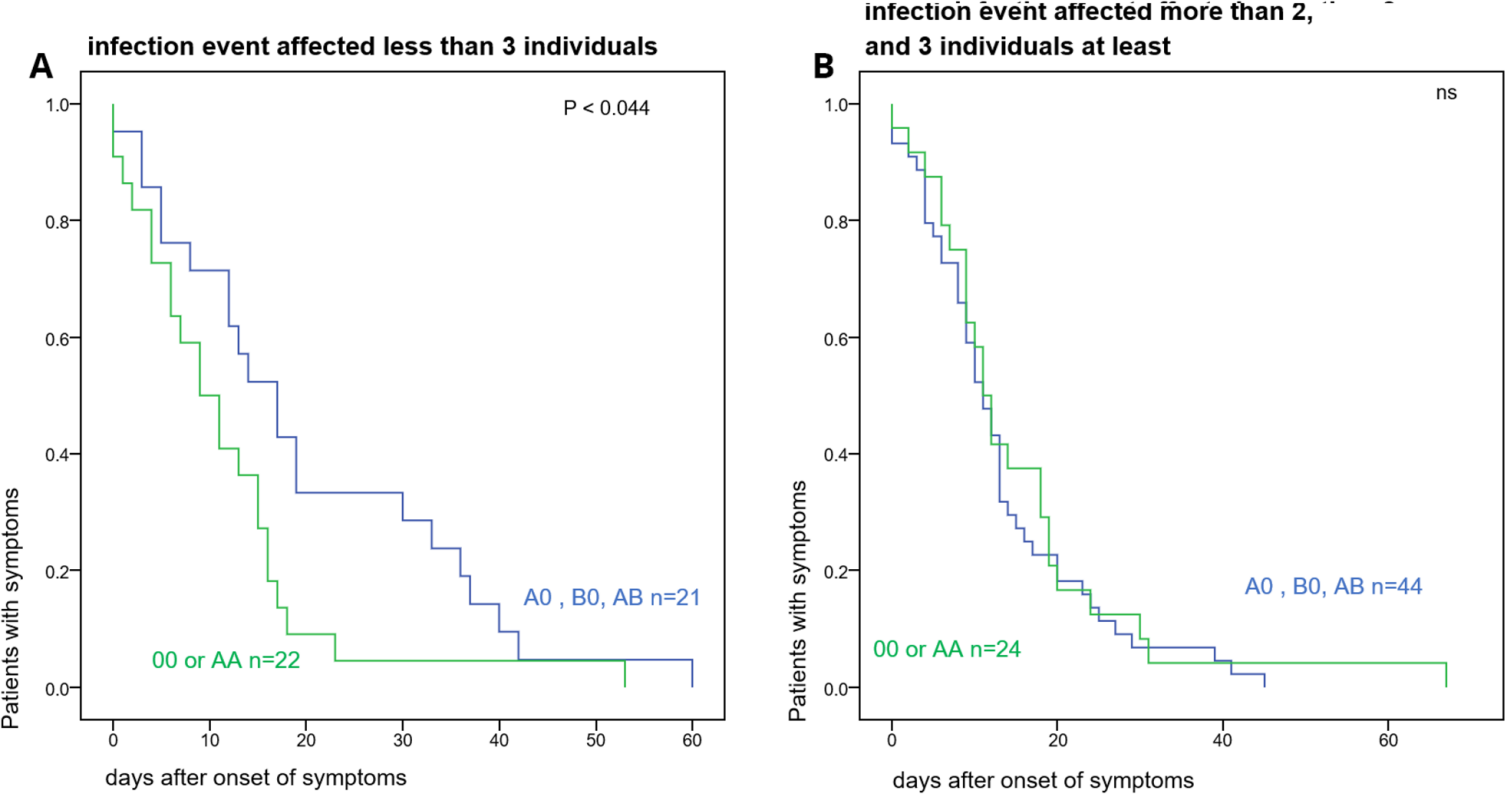
Effect of concomitant infection on the association of AB0 blood group heterozygosity with prolonged disease duration. **A** shows the disease duration in 43 individuals in whom infection event affected less than 3 individuals. **B** shows disease duration in 68 individuals in whom the infection event affected at least 3 individuals

**Fig. 7:**
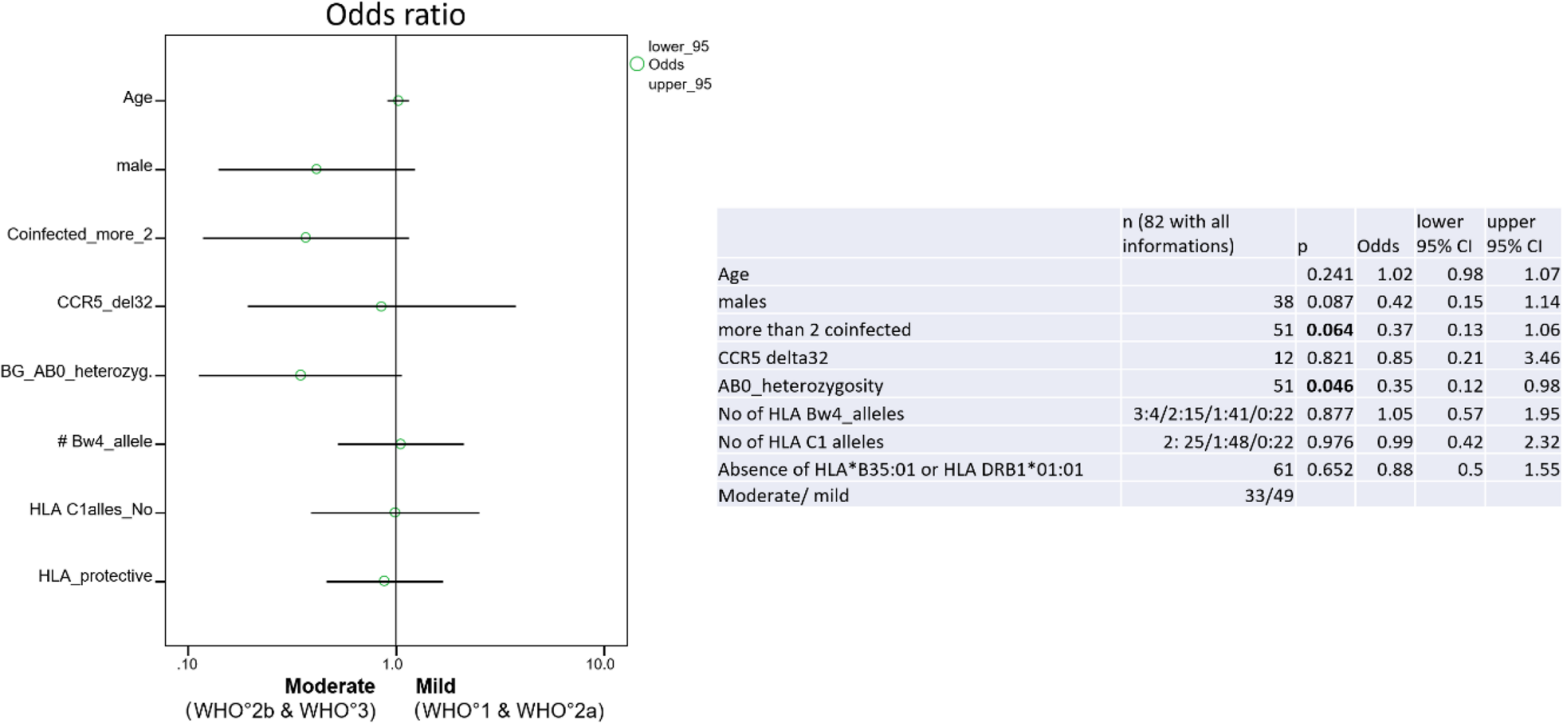
Association of AB0 blood group heterozygosity with development of WHO° 2b and WHO° 3 disease. A logistic regression analysis included as covariates gender, age, multispreading (whether infection event affected at least 3 individuals), ‘‘protective “ HLA alleles (as defined on the Results section), the further class I HLA alleles C2 and HLA-Bw4 (included on this analysis for their binding to distinct killer-cell immunoglobulin-like receptors (KIRs) expressed by natural killer-cells), the heterozygous CCR5 delta 32 mutation and AB0 blood group allele heterozygosity. Odds ratios and their confidence intervals for individuals with COVID 19 WHO° 1 to WHO° 3 are shown

The 22 AB0 homozygous individuals had a significantly (*p* < 0.044) shorter disease duration than the 21 heterozygous individuals, when the infection event affected less than 3 individuals (Fig. 6a). AB0 heterozygosity was associated with a higher risk of a more severe disease (OR 2.95, 95%CI 1.06 to 8.19, *p* < 0.046). Interestingly, when 3 or more people were co-infected, the risk for more severe disease tended to increase to a similar extent (“super spreading event” putatively associated with higher virus load) (Fig. 7).

**Fig. 8:**
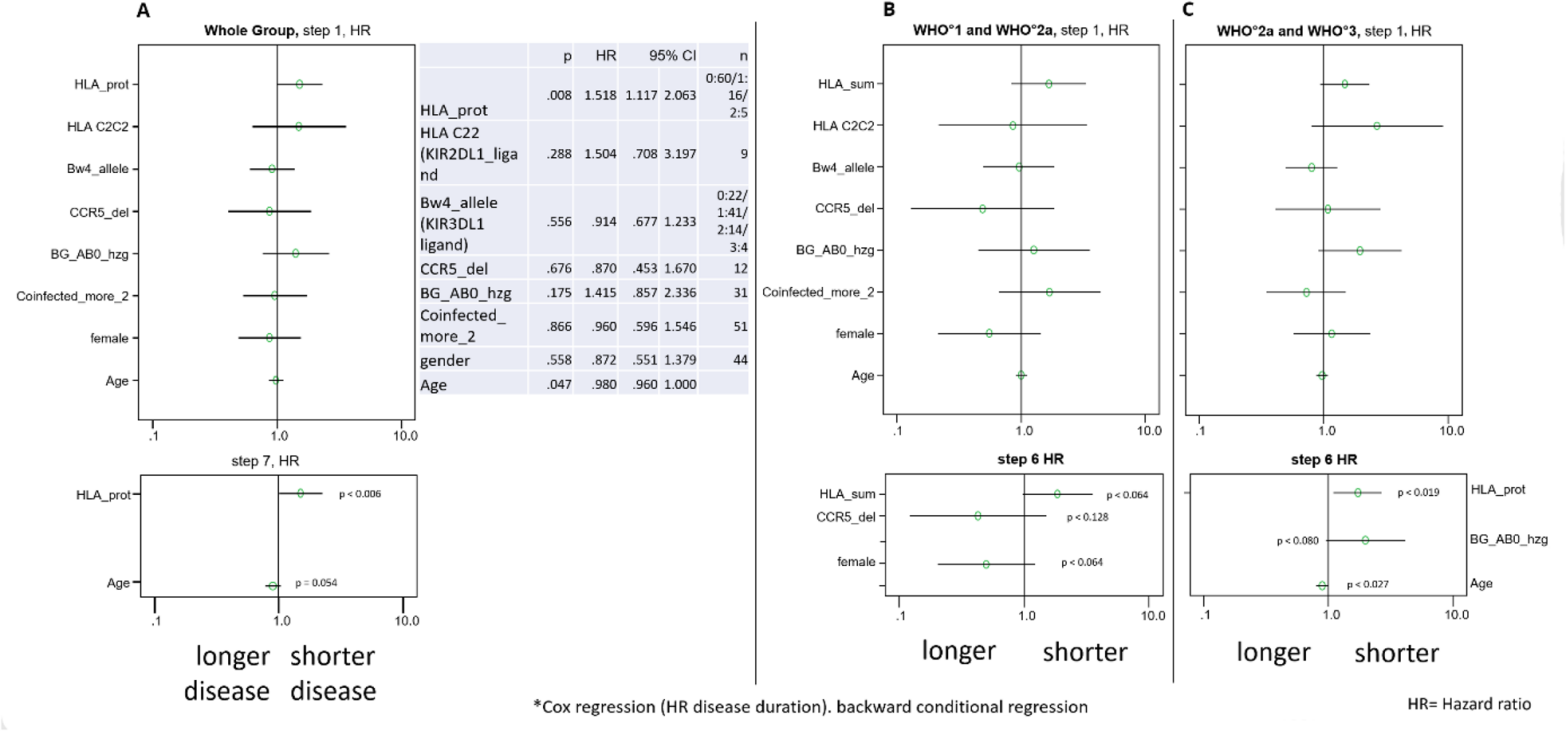
HLA alleles as most informative prognostic biomarkers of disease duration. A Cox regression analysis included as covariates gender, age, number of ‘‘protective “ HLA alleles, HLA encoded NK-KIR ligand for KIR2DL1/ KIR2DS1 (HLA-C2) and KIR3DL1/KIR3DS1 (HLA-Bw4), heterozygous CCR5 delta 32 mutation, as well as AB0 blood group allele homozygosity. **A** The results of a Cox regression analysis performed in 81 individuals for whom all the listed information is available. **B** shows the results of a Cox regression analysis performed in 51 individuals with WHO° 1 and WHO° 2a disease. **C** The results of a Cox regression analysis performed in 30 individuals with WHO° 2a and WHO° 3 disease. Hazard ratio for disease duration and respective 95% confidence interval is shown for the first step and the last step after backward conditional regression

In a Cox regression analysis including the covariates gender, age, the ‘‘protective “ HLA alleles (as defined above), the class I HLA alleles Bw4 and C2 (introduced into this analysis, in addition, as they bind to distinct KIRs thought to play key roles in the first line innate antiviral immune defense by natural killer cells [26]), heterozygous CCR5 delta 32 mutation, as well as AB0 blood group allele homozygosity, ‘‘protective “ HLA alleles emerged as the strongest predictors of short disease duration (HR 1.5, 95%CI: 1.1–2.1) (Fig. 8a–c). In the subgroup of 51 individuals suffering from WHO° 1 and WHO° 2a (mild) disease, “protective” HLA alleles, absence of CCR5 delta 32 mutation and male gender were predictors of shorter disease duration (Fig. 8b).

## Discussion

COVID-19 clinical courses are highly variable between patients, with the immune mechanisms underlying this variability still incompletely understood. Understanding whether and to which extent this clinical variability is associated with given patient’s immunogenetic background characteristics is of utmost importance for a variety of reasons: first, awareness of genetically associated risk may inform individual patient’s prognosis and, in consequence, prophylactic and therapeutic strategies. Second, a deeper understanding of the association of immunogenetics and adaptive mastery of infection may allow to better assess convalescent plasma donations for their protective capacity in the future. Third, with SARS-CoV-species specifically and corona viridae in general undergoing continuous evolution, it may become critical to have established the relevance of immunogenetic background characteristics in the current pandemic to have access to better targeted and more effective early intervention strategies in future outbreaks. Last not least, the S-protein targeted vaccination programs currently launched worldwide immediately raise the question whether and to which extent protective vaccination responses may be impacted by patient’s immunogenetic backgrounds, responding to a novel viral antigen eliciting both de novo adaptive immune responses and expanding pre-existent cross-reactive adaptive immunity.

The current study offers partial answers and may thus help to chart out potential directions of future investigations providing conclusive answers, alongside ongoing vaccination campaigns.

First, this observational study in 157 adult convalescent COVID-19 patients affirms that total anti-S IgG titers in response to SARS-CoV-2 infection are highly variable interindividually and are not adequate to reliably assess the level of antiviral immune protection in a given patient. Our study found patients immediately upon recovery from infection without detectable (16%) or very low (8%) anti-S IgG levels. We interpret this finding to indicate a pre-dominantly cellular antiviral immune response or better protective anti-S IgG epitope specificities in these patients. Second, we observed a partial dissociation of anti-S IgG titers and the capacity of virus neutralization, suggesting that total anti-S IgG levels may represent mixtures of both protective and non-protective epitope specificities. The systematic dissection of epitope specificities may deliver assay methodologies exclusively assessing antivirally protective anti-S antibodies in the future.

The above data are in line with a recently reported study by Klein et al. [22] in a similar convalescent cohort showing that total anti-S IgG titers are not accurate for confirming samples that are negative for neutralizing antibody responses. The assessment of this earlier study did not change when total anti-S IgG titers were replaced by IgG antibody titers directed to the S1 or to the receptor binding domain of the S protein [22].

Importantly, the current study sheds new light on the association of immunogenetic background characteristics with the clinical course of COVID-19 as well as with the immune response thereto. Based on biologic functions of CCR5 established already, our data allow to outline an immune-mechanistically consistent interpretation of the role of the CCR5 delta32 deletion mutant in COVID-19. Based on univariate analyses, we observed that heterozygous carriers of this near-loss-of-function mutant suffered from disease of prolonged duration rather than more severe disease. Significantly reduced anti-S IgA levels in mutant carriers suggested that the nasopharyngeal humoral adaptive immune response is reduced in these patients, in line with the established biologic functions of CCR5 as a chemotactic receptor, and also in line with a recent epidemiological study suggesting increased susceptibility of CCR5 delta 32 mutant carriers to SARS-CoV-2 infection [4]. We neither observed a difference in anti-S IgG level nor in disease severity between patients carrying mutant and wild-type CCR5.

Of note, the proportion of heterozygous carriers of the CCR5 delta 32 mutant within the current study population was small (*n* = 23) and homozygous carriers were not represented. This may have impacted the strength of associations observed and certainly contributed to the fact that hierarchical multivariate (Cox regression) analyses did not confirm the association observed by univariate analyses. It remains to be seen, in consequence, whether future studies also representing homozygous carriers of the CCR5 delta 32 mutation as well as larger patient cohorts will confirm the association between CCR5 delta 32 mutant and COVID-19 disease observed in the current exploratory study.

Most importantly, our data identify two HLA class II alleles (DRB1*01:01 and DRB1*03:01) and one HLA class I allele (B*35:01) associated with reduced COVID-19 duration compared to patients not carrying these alleles. Notably, the association observed with “protective” HLA alleles is stronger than the association with age, i.e., the parameter generally accepted to be best predictive of COVID-19 clinical risk. Furthermore, one of the HLA class II alleles (DRB1*03:01) has been identified as partially protective in earlier SARS-CoV-1 as well as SARS-CoV-2 studies before [12–14, 27]. Intriguingly, “in silico” analyses assign high cumulative calculated HLA-allele affinity scores to the three protective HLA alleles identified by the current study, adding to the biological plausibility of the association observed. The identification of protective alleles belonging to both HLA class I and class II is in line with the general immune-mechanistic concept that both CD4 helper cells (interacting via HLA class II with antigen presenting cells) and CD8 cytotoxic cells (interacting via HLA class I) play important roles in SARS-CoV-2 antiviral immune responses [18]. Of note, we did not find any similar association of COVID-19 duration (or severity) with class I HLA alleles (C2, Bw4) binding to distinct killer-cell immunoglobulin-like receptors thought to play key roles in the first-line innate antiviral immune response. Our analyses also identified two HLA alleles (HLA class II DQB1*03:2 und HLA class I B*15:01) associated with trends towards prolonged duration of disease, whereby B*15:01 had been noted to be associated with increased susceptibility to severe disease before [28].

The HLA associations reported here may be considered complementary to the earlier identification of multiple SARS-CoV-2 HLA class I and HLA class II peptides as potential T cell epitopes in COVID-19 convalescent patients, on the one hand [10], and the identification of CD4 and CD8 T cell populations responsive to SARS-CoV-2 HLA class I and HLA class II predicted peptide “megapools”, on the other [29]. The identification of distinct “protective” HLA alleles as reported here, may inform these analyses and facilitate the identification and prioritization of SARS-CoV-2 peptides of particularly high protective capacity.

Of importance, our study found protective HLA alleles not associated with increased yet rather with decreased total anti-S IgG antibody levels, once again illustrating the lack of utility of this measure as a surrogate of antiviral immune protection.

The dissociation of total anti-S IgG levels and disease controlling immune response, as observed in the current study, may be of far-reaching implication for the future evaluation and therapeutic practice of convalescent plasma therapy.

Our data strongly suggest, in line with a recent study [22], that exclusive reliance on total anti-S IgG titers does not adequately account for the inter-individual variability of protective versus less protective anti-S antibody idiotype titers in a given sample. The heterogeneity of thus-defined convalescent plasma samples in terms of their immune-protective capacity may have contributed to variable levels of benefit generally observed from convalescent plasma therapy, in the past [30].

The analyses of blood group antigens in the current study are in line with earlier observations that individuals with the phenotype A—where AB0 heterozygosity denotes to 90% of these individuals—is associated with more severe clinical courses of COVID-19. In fact, AB0 heterozygosity emerged as the only genetic parameter significantly associated with more severe (rather than prolonged) disease, in our analyses. However, case numbers in our study are too small to rule out other genetic associations given the interindividual heterogeneity of clinical course and large spectrum of possible confounders.

In summary, the current study identifies immunogenetic background characteristics, specifically distinct HLA genotypes, as being strongly associated with naïve host’s mastery of infection with the SARS-CoV-2 neo-virus, with this association being even stronger than the association with patients’ age.

This study is associated with a number of limitations. This is an observational, non-interventional, uncontrolled study of exploratory design adequate for hypothesis generation yet neither designed nor powered for confirming prospectively defined hypotheses. The population deliberately consisted of patients with mild to moderate rather than severe disease to focus analyses on effective antiviral immune response patterns not impacted by fundamentally aberrant virus-induced immune phenomena which characterize COVID-associated acute respiratory syndrome and life-threatening disease. This dissection was introduced because it is reasonable to assume that serious COVID-19 is dominated by virally determined molecular and cellular immune mechanisms which are distinct from those relevant to preemptive mastery of infection, as is the focus of this investigation. A limitation remains, nevertheless, that patients with WHO grade ≥ 4 are not represented in this study. Even though data collection and analysis followed a prospectively defined statistical and analysis plan, blood sampling reflected routine practice and did not follow a pre-specified sampling schedule. To control for the testing of multiple variables, key conclusions were subjected to hierarchical Cox regression analysis. Further investigations in larger, prospectively defined populations are needed.

## Conclusion

The current study shows that the presence of certain HLA alleles is of even stronger association with reduced disease duration of mild and moderate COVID-19 than age or any other potential risk factor assessed. Univariate analysis uncovered an association of heterozygous CCR5 delta 32 mutation with prolonged disease duration. Cox regression analysis of these multivariate data appears to grade the influence of HLA-genotypes genotypes over the CCR5 delta 32 status for the duration of the disease. Severity of the disease seems to be strongly associated with the AB0 blood group allele status of the patients.

## Supplementary Information

**Additional file 1.** Additional figures.

## Data Availability

All data and materials can be accessed via CM and FM.

## Abbreviations

CCR5: C–C chemokine receptor type 5
CD 4: Cluster of differentiation 4
COVID-19: Coronavirus disease 2019
CP: Convalescent plasma
e.g.: Exempli gratia
ELISA: Enzyme-linked immunosorbent assay
Ig: Immunoglobulin
IL: Interleukin
IQR: Inter quartile range
MCP: Monocyte chemo-attractant protein
PCR: Polymerase chain reaction
SARS-CoV-2: Severe acute respiratory syndrome coronavirus 2
WHO: World health organization
