# Supplementary material for: Association of HLA genotypes, AB0 blood type and chemokine receptor 5 mutant CD195 with the clinical course of COVID-19
Source: Eur J Med Res. 2021 Sep 16;26:107. doi: 10.1186/s40001-021-00560-4 (PMC8444184; doi:10.1186/s40001-021-00560-4)
Supplement: Supplementary file 1 — Additional file 1. Additional figures. [file 40001_2021_560_MOESM1_ESM.docx]

**
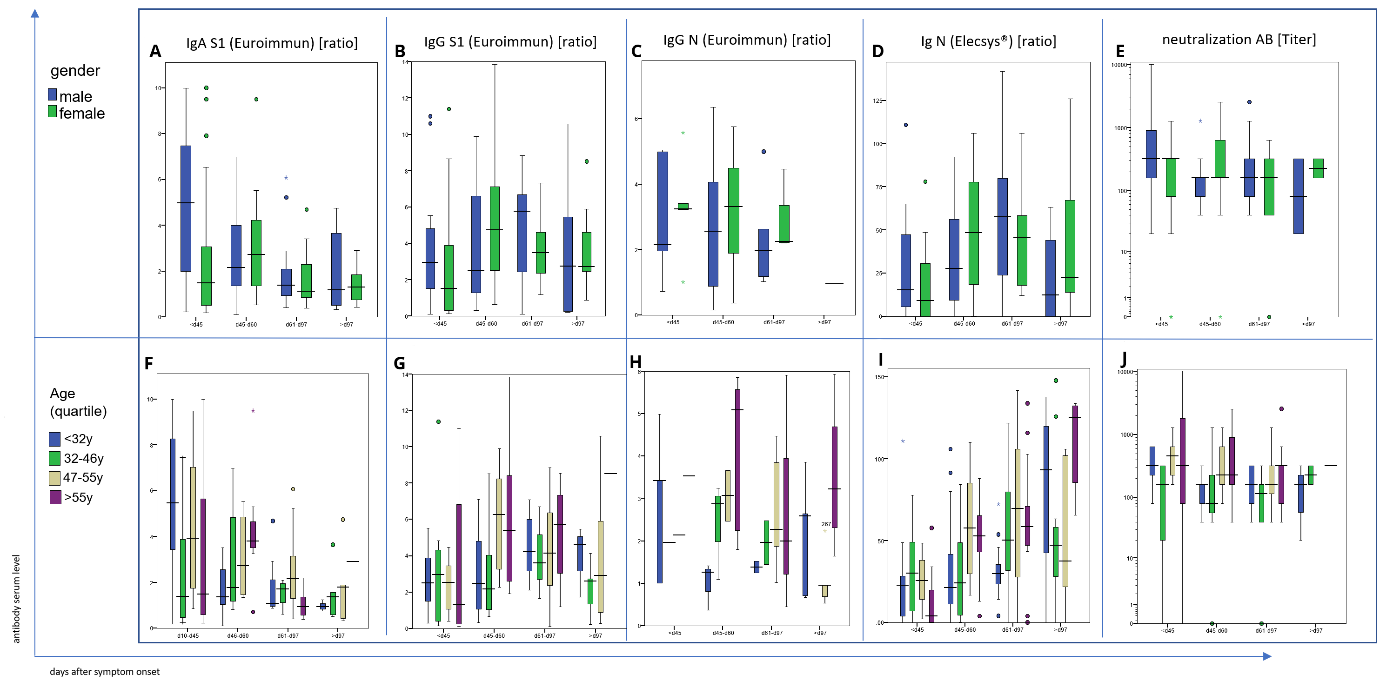
**

**Figure S1: Lack of association of gender and age with** **SARS-CoV-2 viral protein-specific antibody levels determined at the timepoints when the tested individuals entered the study,**

SARS-CoV-2 viral protein-specific antibody levels were determined as described in the legend of Figure 1 in 78 males (blue) and 79 females (green) **Panel** (**A** - **E**) Individuals’ age range from 20 to 77 for male and 20 to80 for female. In the bottom **panel** (**F** - **J**) age is divided into 4 quartiles (quartile split). Blue indicates age from 20 to 31., green 32 to 46y, gold 47 to55y and magenta older than 55y.

Time following symptom onset was divided into quartiles (d10-d45, d46-d60, d61-d97, > d97; it ranged between d10 and d120).

**
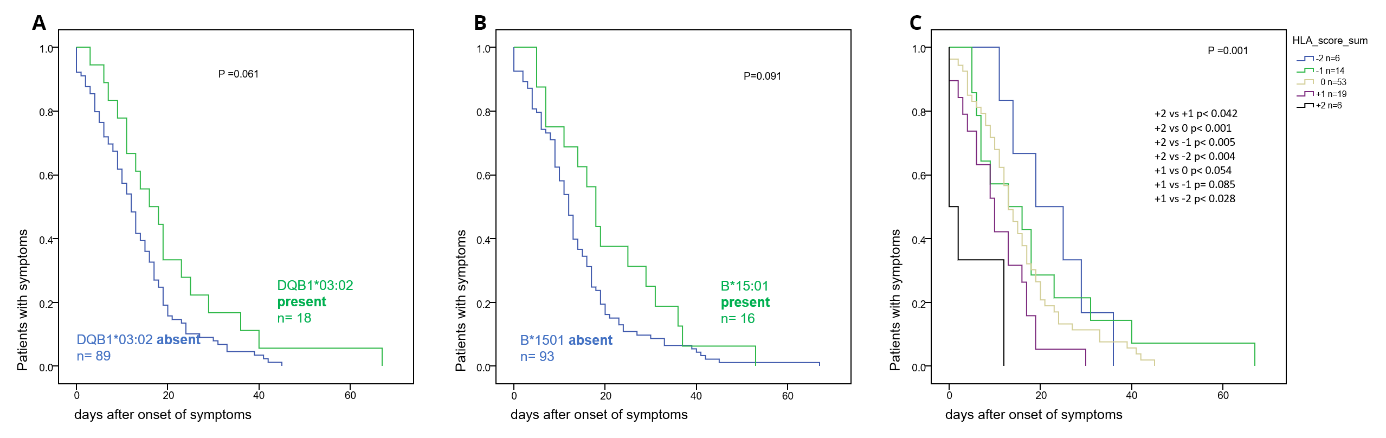
**

**Figure S2: Association of HLA-DQB1*03:02 and/or HLA-B*15:01 allele expression with longer COVID19 disease duration**

**Panel A** shows the disease duration related to HLA DQB1*03:02 expression. **Panel B** shows the disease duration related to HLA-B*15:01 expression. **Panel C** shows the disease duration related to expression of “protective” and “non protective” HLA-alleles. The HLA score was calculated by subtracting 1 from 0 for the expression of each of the “non protective” HLA-B*15:01 or HLA- DQB1*03:02 alleles and adding 1 for the expression of each the “protective” HLA-DBR1*01:01 or HLA- B*35:01 alleles. The possible range of the score is -2 to 2. Two of the 15 individuals with a score of -1 co-expressed two “non protective” HLA alleles and one “protective” HLA allele. One of the 53 individuals with a score of 0 express one “protective” and one “non protective” HLA allele.
